# Science of Hypnotism

**Published:** 1887-10

**Authors:** 


### The Science of Hypnotism.

We have heard a great deal of late years of that peculiar mental condition known as hypnotism, and the attention devoted to it in the medical world has been gradually increasing, thanks to the initiative of the eminent professor of the Salpetriere. The study of the subject has so far developed as to warrant—at any rate in the opinion of the editors—the publication of a monthly journal, the first number of which has just appeared. The *Revue des Sciences Hypnotiques* has for its object the generalization of the knowledge we possess of this ill-understood condition, and to aid in further systematic observation and research. It seems very possible that the subject will in future acquire an importance of which we can hardly form an adequate idea at present, quite apart from its pathological interest. The phenomena of of hypnotism are primarily physiological, though its most striking manifestations are obtained in subjects of a peculiar mental constitution characterized by an abnormal absence of volition. In this country the profession have looked somewhat askance at the study of this psychological problem, and the student would search in vain for an opportunity of familiarizing himself with the modus operandi or its effects. Hitherto the subject has been monopolized by charlatans and mountebanks, a fact which may account to some extent for the distrust shown toward it by medical men. There was a time, however, not so very far removed, when that valuable agent, electricity, was treated similarly, and it needed much courage and resolution to employ it openly. Electricity, however, has passed through its period of probation and is now a recognized and appreciated therapeutic agent. It is possible—we are not now in a position to say probable—that hypnotism may follow in the wake of the other science, and if its manifestations and attributes can only be placed on a sound physiological basis, a great step will have been taken towards its more general appreciation and use.—*Medical Press and Circular.*

